# The Prodrug DHED Delivers 17β-Estradiol into the Retina for Protection of Retinal Ganglion Cells and Preservation of Visual Function in an Animal Model of Glaucoma

**DOI:** 10.3390/cells13131126

**Published:** 2024-06-29

**Authors:** Ammar Kapic, Khadiza Zaman, Vien Nguyen, George C. Neagu, Nathalie Sumien, Laszlo Prokai, Katalin Prokai-Tatrai

**Affiliations:** Department of Pharmacology and Neuroscience, University of North Texas Health Science Center, Fort Worth, TX 76107, USA; ammarkapic@my.unthsc.edu (A.K.); khadiza.zaman@unthsc.edu (K.Z.); vien.nguyen@unthsc.edu (V.N.); georgeneagu@my.unthsc.edu (G.C.N.); nathalie.sumien@unthsc.edu (N.S.); laszlo.prokai@unthsc.edu (L.P.)

**Keywords:** DHED, 17β-estradiol, glaucoma, contrast sensitivity, optomotor response, visual acuity, neuroprotection, retina proteomics

## Abstract

We report a three-pronged phenotypic evaluation of the bioprecursor prodrug 10β,17β-dihydroxyestra-1,4-dien-3-one (DHED) that selectively produces 17β-estradiol (E2) in the retina after topical administration and halts glaucomatous neurodegeneration in a male rat model of the disease. Ocular hypertension (OHT) was induced by hyperosmotic saline injection into an episcleral vein of the eye. Animals received daily DHED eye drops for 12 weeks. Deterioration of visual acuity and contrast sensitivity by OHT in these animals were markedly prevented by the DHED-derived E2 with concomitant preservation of retinal ganglion cells and their axons. In addition, we utilized targeted retina proteomics and a previously established panel of proteins as preclinical biomarkers in the context of OHT-induced neurodegeneration as a characteristic process of the disease. The prodrug treatment provided retina-targeted remediation against the glaucomatous dysregulations of these surrogate endpoints without increasing circulating E2 levels. Collectively, the demonstrated significant neuroprotective effect by the DHED-derived E2 in the selected animal model of glaucoma supports the translational potential of our presented ocular neuroprotective approach owing to its inherent therapeutic safety and efficacy.

## 1. Introduction

One of the characteristic pathological features of many blinding eye diseases such as glaucoma and diabetic retinopathy is the irreversible neurodegeneration of the retina [1,2,3]. Glaucoma is the second leading cause of blindness worldwide and it comprises several different forms of optic neuropathies with various etiologies [4,5]. Approximately 60% of those impacted are women, but there is a significant number of men also affected by the disease considering that currently over 60 million people suffer from a spectrum of glaucoma worldwide [6,7]. For the development and progression of most glaucoma, chronically elevated intraocular pressure (IOP) is an established and the only modifiable risk factor [8]. The disease destroys the retinal ganglion cells (RGCs) and their optic nerve-forming axons gradually, which over time may lead to vision loss despite significant IOP reduction [8,9,10,11]. Therefore, the development of pharmacological agents for retina neuroprotection specifically targeting the prevention or amelioration of glaucomatous neurodegeneration would be tremendously beneficial to overcome this pressing unmet medical need [1,12,13,14].

Glaucomatous neurodegeneration involves apoptosis, compromised anterograde and retrograde axoplasmic transport, ischemia, mitochondrial dysfunction, oxidative stress, inflammation, autophagy dysregulation, amyloid-β/τ pathology, altered ubiquitin signaling, and many other incompletely understood mechanisms [14,15,16,17,18,19]. Such a multi-faceted process makes target-based drug development enormously challenging [20,21]. On the other hand, phenotypic drug discovery has experienced a recent revival owing to its potential to enhance our understanding of complex diseases and their promise of delivering first-in-class drugs [22,23], especially when higher-order preclinical evaluations in small animal models are available [24,25]. Ultimately, vision rescue is the goal of therapy development focused on the prevention of retinal neurodegeneration [26]. Therefore, a rapid behavioral assessment of visual function using the optomotor test, which does not require prior training of the animals [27], in combination with an established rodent model of glaucoma [25], has high translational merit [28,29,30].

**Figure 1:**
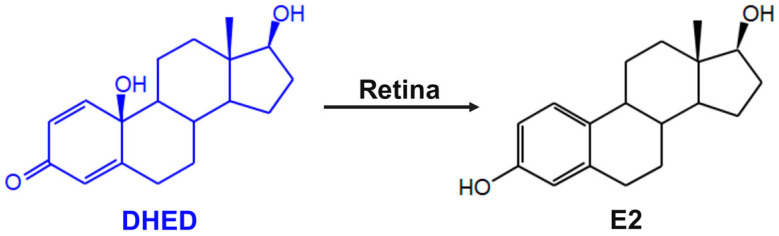
Bioactivation of the bioprecursor prodrug DHED to the neuroprotective 17β-estradiol (E2) in the retina upon topical administration [32,33].

Based on optomotor response (OMR), we report here a phenotypic evaluation of the topically administered bioprecursor prodrug 10β,17β-dihydroxyestra-1,4-dien-3-one (DHED, Figure 1) that selectively delivers 17β-estradiol (E2, Figure 1) to the site of action [31,32,33] for neuroprotection of the retina to halt its glaucomatous neurodegeneration in a rat model of the disease [34]. By delayed genomic and rapid non-genomic mechanisms acting in concert, E2 has been shown to possess profound and broad-spectrum neuroprotective effects to offset practically all suggested critical contributors of neuronal damage in the retina, such as inflammation, oxidative stress, and excitotoxicity [35,36]. However, E2 eye drops were shown to expose the periphery to the hormone eliciting unwanted side effects [28,32,33]. On the other hand, topical DHED treatments produced large E2 concentrations in the male and female rodent retinas due to the site-specific NADP(H)-dependent DHED-to-E2 metabolism (Figure 1) and thus, without increasing circulating E2 [32,33] after a 3-week once daily (q.d.) treatment. 

The rodent OMR-based behavioral evaluation of vision [27,28,29,30,37] can also be augmented by follow-up examinations of retinal structural changes involving the RGCs and optic nerve through postmortem histological analyses and/or through confirmation of selected surrogate endpoints such as in situ tissue staining that detects apoptotic cell death [28]. Nonetheless, methodological concerns about retinal immunohistochemistry [38,39] and limitations of apoptosis assays to measure cell death have also been recognized [40]. Therefore, drug discovery and early-stage development will benefit from introducing disease-indicating molecular biomarker panels relying on preclinical models [41]. 

Retina proteomics has been emerging as an enabling methodology for this purpose [42]. Untargeted exhaustive analyses, however, are considered associative with low specificity and sensitivity [43], albeit they are essential for the assembly of a targeted panel of preclinical biomarkers that can be measured specifically and reproducibly as surrogate endpoints of experimental interventions against retinopathies [44]. In this context, we have recently reported ocular hypertension (OHT)-associated changes in the expressions of several proteins by mass spectrometry-based targeted proteomics focusing on the Morrison model of glaucoma [34] in male retired-breeder Brown Norway (BN) rats [45]. This panel of preclinical biomarker candidates broadly represented cell death and survival, neurological disease, ophthalmic disease, organismal injury and abnormalities when assembled into a protein interaction network. Top canonical pathways included mitochondrial dysfunction, S100 protein family signaling, and chaperone-mediated autophagy signaling. In the present studies, for the first time, we have complemented the behavioral evaluations of visual function based on the OMR in this glaucoma model while also assessing RGC and axon survivals together with the developed targeted proteomics assay that enabled the survey of our previously proposed surrogate endpoints [45] upon utilizing the DHED-based ocular neuroprotection approach.

## 2. Materials and Methods

### 2.1. Chemicals and Reagents

DHED was prepared in our laboratory, as reported before [46]. Reagents for the synthesis were purchased from Millipore Sigma (St. Louis, MO, USA). Sequencing-grade trypsin was from Promega (Madison, WI, USA). Donkey serum and primary rabbit antibodies against RNA-binding protein with multiple splicing (RBPMS) were from GeneTex (Irvine, CA, USA). Sodium cacodylate buffer was supplied by Electron Microscopy Sciences (Hatfield, PA, USA). Alexa Fluor 488 donkey anti-rabbit secondary antibody, Fluoromount-G with Dapi, paraformaldehyde, glutaraldehyde solution, and chromatographic solvents (Optima^®^ LC/MS grade) were acquired from Thermo Fisher Scientific (Waltham, MA, USA). Agents used for anesthesia and euthanasia were purchased from Covetrus (Fort Worth, TX, USA).

### 2.2. Animals 

All procedures conformed to the ARVO Statement for the Use of Animals in Ophthalmic and Vision Research. Before the initiation of the studies, all protocols were approved by the Institutional Animal Care and Use Committee at the University of North Texas Health Science Center (approval numbers: #IACUC-2023-0012 approved on 05/02/2023 and #IACUC-2022-0028 approved on 11/04/2022). Retired breeder BN male rats (8–10 months old, weighing 200–225 g) were purchased from Charles Rivers Laboratories (Wilmington, DE, USA). Animals were housed under standard conditions with ad libitum access to food and water.

### 2.3. IOP Measurements and Elevation to Induce Glaucoma 

IOP was measured in awake and hand-held animals by a TONOLAB tonometer (iCare USA, Inc., Raleigh, NC, USA) longitudinally throughout the duration of this study, always between 10–11 a.m., local time. Pressure readings (4 to 6 at each point) were recorded and averaged to give one IOP measurement per eye. These readouts for both eyes within the same animal were then averaged and taken as one IOP measurement [47]. Baseline IOP measurements were taken in each animal used in our studies with a cumulative average of 14.3 ± 0.8 mmHg (mean ± SD). These measures were first taken one week (considered as week 3) prior to the induction of OHT, bilaterally (week 2) as well as right before the animals were anesthetized (75 mg/kg body weight ketamine and 10 mg/kg body weight xylazine, intraperitoneal) for an episcleral injection of 50 µL of 1.8 M saline (n = 9, 18 eyes), as reported before [28]. The cumulative net IOP increase was approximately 50% compared to the baseline IOP, which remained consistent over the study period. This was achieved in about 80% of animals, 6–8 days post-saline injection. Normotensive age-matched (naïve) animals (n = 4, 8 eyes) served as control.

### 2.4. Eye Drop Treatments

The sterilized eye drops contained 0.1% (*w*/*v*) DHED in a saline vehicle containing 20% (*w*/*v*) hydroxypropyl-β-cyclodextrin (HPβCD) [32,33]. The instillation volume was 10 µL. Eye drop treatments were carried out q.d., between 10–11 am, local time, and started at week 0. In the vehicle-treated group n = 5 (10 eyes), while in the prodrug-treatment group n = 4 (8 eyes) animals were used. At the end of the study period, animals were euthanized (100 mg/kg body weight ketamine and 10 mg/kg body weight xylazine, i.p.) and blood was collected by cardiac puncture in a BD Vacutainer (Fisher Sci., Atlanta, GA, USA) to make serum. The eyes were then enucleated immediately followed by a quick dissection to collect the retina, optic nerve, as well as other ocular and non-ocular tissues that were stored at –80 °C until processing. Seminal vesicles (SV) and anterior pituitaries (AP) were also collected, and their wet weights were used as independent markers for the presence of circulating E2 [48,49].

### 2.5. Assessment of Visual Function 

The visual acuity (VA) and contrast sensitivity (CS) were assessed using the OptoMotry system (Cerebral Mechanics, Medicine Hat, Alberta, Canada), as reported before [28]. The testing apparatus comprised a mirrored floor with four computer monitors serving as the walls and a video camera looking down on a raised platform. The OptoMotry 1.7 software was used to adjust the gratings for the virtual cylinder and record the observed head-tracking. Eyes were assessed independently using randomized alternating directions (clockwise or anticlockwise). Before testing, rats were dark-adapted in their cage in the testing room for 1 h. For VA, each successive observation of head-tracking resulted in a progressive increase in frequency following a staircase method which alternated with lower and higher frequencies. When tracking was no longer observed, the test would end, and the highest frequency was considered as the visual acuity. CS was measured at two fixed spatial frequencies (0.103 and 0.272 c/d, where c/d denotes cycles per degree) [28,50]. We calculated CS function as 1/C, where C was the lowest contrast that elicited a response at a particular frequency [51]. Accordingly, when a rat can see at a lower contrast setting, the sensitivity number will appear larger and indicate better visual performance at a particular spatial frequency. Baseline VA and CS values were collected prior to inducing the experimental glaucoma. VA and CS were also assessed in the naïve control group (n = 4, 8 eyes). Studies were conducted by a trained observer who was unaware of the treatment protocol. For each animal, the readouts for the 2 eyes were averaged and counted as one experimental value per animal [47]. 

### 2.6. RGC Quantification 

The experiment was carried out according to a previous report [52] using randomly selected left or right retina from each treatment (n = 4 and 5 from the DHED and vehicle-treated groups, respectively) and the naïve control group (n = 4). Briefly, retinas were washed and then incubated for 1 h in a blocking solution composed of 5% donkey serum and 0.4% Triton X-100 in phosphate-buffered saline (PBS). Subsequently, retinas were incubated overnight with anti-RBPMS diluted in the blocking solution followed by washing with PBS three times and then overnight incubation with the Alexa-Fluor 488 secondary antibody. The secondary antibody was again washed with PBS three times. The retinas were then mounted to the slides and cover slipped using Fluoromount-G mounting media to protect the fluorophore and seal the coverslip. Images were taken at a magnification of 40× using the Keyence BZ-X810 all-in-one fluorescence microscope (Itasca, IL, USA) with the system’s GFP filter (Ex480/40 Em535/50). The area of each region was approximately 0.1 mm^2^ and was randomly selected by a blinded observer. RBPMS-positive cells were counted using the publicly available cell counting plugin ImageJ image processing software [53]. 

### 2.7. Optic Nerve Axon Counts 

Selected optic nerves were extracted and fixed overnight in 2% paraformaldehyde, 2.5% glutaraldehyde solution, and 0.1 M sodium cacodylate buffer. Next, optic nerves were post-fixed with 2% osmium tetroxide solution in 0.1 M sodium cacodylate buffer. The optic nerves were then dehydrated and embedded in Epon resin. Cross sections of the optic nerve were cut using the Leica EM UC7 ultramicrotome (Wetzlar, Germany) at 500 nm, mounted to slides, and stained with 1% paraphenylenediamine (PPD) dissolved in a 50:50 *v*/*v* mixture of methanol/propanol to visualize the myelin [54]. The prepared PPD slides were imaged using the Keyence BZ-X710 microscope (Itasca, IL, USA). A blinded observer took non-overlapping images of the optic nerve sections at 100× magnification. Automated counting of the axons was performed by the AxoNet 2.0 software [55]. The average density per field was calculated by averaging the total axons counted per 100× field, then converted to axon/mm^2^. 

### 2.8. Measurement of Serum E2 

Serum E2 was quantified by our validated liquid chromatography–tandem mass spectrometry (LC–MS/MS) assay using the principles of isotope dilution, as reported before [56,57]. Ultrahigh performance liquid chromatography (UHPLC) separations were carried out on a 5 cm × 2.1 mm i.d. Kinetex phenyl-hexyl column (1.7 µm) from Phenomenex (Torrance, CA, USA) using a Vanquish UHPLC system (Thermo Electron Corporation, Trace Chemical Analysis, Austin, TX, USA). The binary gradient was mixed from 0.1% formic acid (FA) in water and 0.1% FA in acetonitrile to elute the analytes [55]. The triple-quadrupole mass spectrometer (TSQ Quantum Ultra, Thermo Electron Corporation) was operated in positive ion mode with a heated electrospray ionization (ESI) probe and using Xcalibur (version 4.0, Thermo Fisher Scientific, Waltham, MA, USA) data acquisition software. Selected reaction monitoring (SRM) with unit mass resolution for the precursor and product ions was used for quantification. SRM transitions of *m*/*z* 506 → 171 and 512 → 171 were set up for the dansylated- E2, and ^13^C_6_-E2 internal standard, respectively.

### 2.9. Targeted Proteomics 

Protein extraction from the retinas, reduction, alkylation, trypsin digestion, and sample cleanup were conducted according to our previously reported procedures [33,45,58]. Briefly, samples (1 µg/µL protein) were reconstituted in 5% aqueous acetonitrile containing 0.1% FA. For the quantification of the selected panel of OHT-affected proteins, our nano-LC–ESI-MS/MS-based targeted proteomics used the parallel reaction monitoring (PRM) method we reported previously [45]. Samples were run on an LTQ Orbitrap Velos Pro tandem mass spectrometer connected to EASY nanoLC-1000 systems and fitted with an EASY-Spray source (Thermo Fisher Scientific, San Jose, CA, USA) with separations performed on an EASY-Spray column packed with 15 cm × 75 μm i.d. PepMap C18 particles (3 µm). Peptides were eluted at 300 nL/min flow rate with a 2 h binary solvent gradient (from 5% to 40% *v*/*v* acetonitrile in an aqueous eluent containing 0.1% FA) [45]. Full MS/MS scans acquired using 2.0 Th isolation width, 35% normalized collision energy, 30 ms activation time, and nominal mass resolution of the Orbitrap analyzer set to 15,000 (at *m*/*z* 400) were imported into Skyline (version 21.1.0.146, MacCoss Lab Software, University of Washington, Seattle, WA, USA) [59] to extract product ion chromatograms using the built-in PRM mode of the program. Parameters for this procedure included precursor charge (z) of 2 and product ion charges of 1 and 2 with b- and y-type fragment ions considered at 0.5 Da mass tolerance (Table S1). Group comparisons were performed using the transitions reported by Skyline’s descriptive statistics calculated for each group and all outcomes. Specifically, the reported abundances were used for relative quantitation by obtaining fold changes calculated from all technical replicates of the samples for each peptide of the chosen PRM panel (Table S1).

### 2.10. Statistical Analyses 

Data are represented as mean values ± standard error mean. Statistical analysis was conducted using Origin (OriginLab, Northampton, MA, USA) with α value set to 0.05. For IOP measurements, a two-way repeated measures analysis of variances (ANOVA) with a post-hoc Tukey test was used to compare the changes in IOP using time and treatment group as factors. For CS and VA, one-way ANOVA with a post hoc Tukey test was used to compare treatment groups at weeks 3 and 12 with the naïve control group. RGC and axon survivals were compared using a one-way ANOVA with a post hoc Tukey test. Finally, statistics for targeted proteomics were achieved by one-way ANOVA followed by post hoc Tukey–Kramer test. 

## 3. Results

### 3.1. Impact of Topical DHED Treatments on OHT-Induced Visual Decline Based on OMR, RGC and Axonal Loss

**Figure 2:**
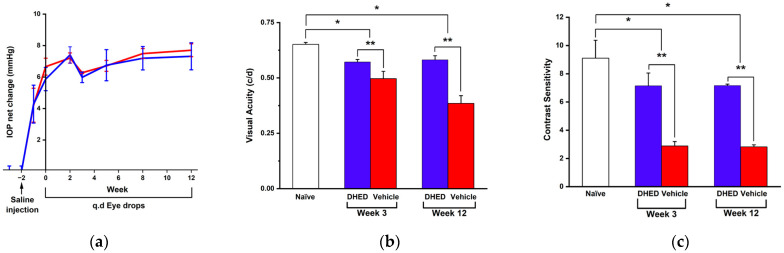
Neuroprotective effect achieved by DHED-derived E2 under OHT measured as visual acuity (VA) and contrast sensitivity (CS) based on the optomotor response (OMR) of the animal. (**a**) Net IOP changes compared to normotensive naïve control animals after vehicle (20% *w*/*v* HPβCD in saline, red line, n = 5) or DHED (0.1% *w*/*v* in vehicle, blue line, n = 4) administration, q.d. topically as eye drops starting at week zero for 12 weeks. Week –2 was the time of the initiation of IOP elevation by saline injection into an episcleral vein. (**b**) DHED-derived E2 halted OHT induced a gradual decrease in VA (blue bars) and (**c**) rapid decrease in CS at 0.272 c/d spatial frequency (blue bars) compared to the vehicle-treated animals (red bars). Data are given as average ± SEM. * *p* < 0.05 compared to naïve control (n = 4); ** *p* < 0.05 compared to DHED-treated group (one-way ANOVA followed by post-hoc Tukey’s multiple comparison test).

First, the experimental model of glaucoma was created by hyperosmotic (1.8 M) saline injection into an episcleral vein of the rat eye [28,34]. The IOP was monitored longitudinally. The baseline value (14.4 ± 0.3 mmHg) was established before the saline injection to initiate OHT for all animals involved in this study. By the 2nd week post-saline treatment, approximately 45–50% elevation of IOP was achieved in relation to the baseline that remained sustained throughout the duration of this study. At this point considered as week zero (Figure 2a), OHT animals started receiving q.d. eye drop treatments for 12 weeks. The eye drops contained either 0.1% (*w*/*v*) DHED in a saline vehicle containing 20% (*w*/*v*) HPβCD or the vehicle alone, as reported before [32,33].

Figure 2a also shows that neither DHED nor the vehicle eye drops significantly altered the IOP elevation. Two-way repeated measures ANOVA analysis also revealed that at any time point during this study, there were no statistically significant differences in IOPs between the two treatment groups. Hence, DHED that produces a large E2 concentration in the retina [31,32,33] had no IOP-lowering effect. However, a significant preservation of visual function was detected upon administering this prodrug to the OHT animals, as shown in Figure 2b,c.

Visual acuity (VA) was monitored after 3 [27,32,33] and 12 weeks of q.d. eye drop treatments, respectively, using the OMR of the rat. In the DHED-treated group, VA retained approximately 90% of what was measured in the normotensive (naïve) control animals (0.642 ± 0.023, Figure 2b). At the same time, a gradual worsening of VA could be observed in the vehicle-treated group, and by the end of the study period (12 weeks), this measure dropped to 0.385 ± 0.035 c/d (Figure 2b) owing to the sustained IOP elevation (Figure 2a). Alongside, a drastic difference in contrast sensitivity (CS) at the given 0.272 spatial frequency [50] could also be recognized between the two treatment groups. As shown in Figure 2c, there was approximately a 70% drop in CS compared to the naïve control (9.1 ± 1.3) even after a 3-week continuous OHT, when animals received eye drops containing the vehicle alone. In the DHED-treated group, however, CS decreased by around 15% only compared to the normotensive control, indicating significant neuroprotection against visual dysfunction brought about by OHT-triggered RGC damage. A similar trend was also observed when CS was evaluated at 0.103 c/d spatial frequency [27]. This measure dropped to 2.7 ± 0.3 in the vehicle-treated group at 12 weeks in sharp contrast relative to the naïve control group (7.4 ± 0.9), while in the DHED-treated group, CS remained over 80% of that of control at the end of the study period.

**Figure 3:**
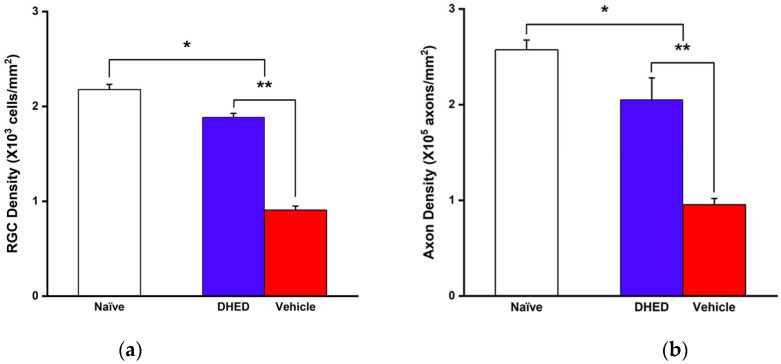
DHED-derived E2 prevents OHT-triggered RGC death and axon loss. (**a**) Mean RGC density in the vehicle group (n = 5, red bar) was significantly reduced when compared to naïve control (n = 4, open bar); however, E2 formed from the prodrug significantly preserved RGCs (n = 4, blue bar). (**b**) Axon density in optic nerves from vehicle-treated group (n = 5, red bar) was significantly lower than in the naïve control (n = 4) and the DHED-treated group (n = 4), respectively. Statistics are one-way ANOVA followed by post hoc Tukey’s multiple comparisons test. * *p* < 0.05 compared to naïve control group; ** *p* < 0.05 compared to DHED-treated group.

Collectively, both outcomes of the OMR-based behavioral evaluations of vision (Figure 2b,c) confirmed the benefit of DHED-derived E2 upon topical administration of the prodrug to counteract OHT-induced RGC damage, thus, for the preservation of RGC viability and function. At the end of the 12-week observational period, RGC survival was evaluated by counting on the flat-mount RBPMS-immunostained whole retina [52]. Optic nerve axon counting was performed on PPD-stained cross sections [53]. As expected, there was a statistically significant difference in both readouts between the two treatment groups (Figure 3a,b). In the retinas of the animals receiving vehicle eye drops, there were around 60% less RGCs (908 ± 80 cells/mm^2^) than in the naïve control (2177 ± 110 cells/mm^2^) by the end of the 12-week treatment period, owing to the detrimental effect of sustained OHT (Figure 2a). In contrast, RGC density was 1877± 82 cells/mm^2^ in the DHED group. Thus, DHED-derived E2 halted RGC death because twice as many RGCs remained in the retinas of the prodrug-treated animals compared to those in animals receiving the vehicle alone (908 ± 40 cells/mm^2^). 

Consistent with RGC survival in the DHED group (Figure 3a and Figure S1), axon density in the optic nerve cross sections was also preserved compared to the vehicle-treated group (Figure 3b and Figure S1). In fact, like with RGCs preservation, a significantly higher axon density was present in the prodrug-treated group (205,255 ± 22,791 axon/mm^2^) than without neuroprotective intervention, thus, treating the animals with the vehicle only under continuous OHT (95,432 ± 6545 axon/mm^2^). Overall, these findings further substantiate the OMR-based behavioral assessments in terms of the substantial preservation of visual function in the OHT animals by the topical DHED treatment (Figure 2b,c).

As DHED is an inert prodrug that efficiently metabolizes to the neuroprotective E2 in the retina (Figure 1) [32,33], therapeutic safety considerations are needed upon sub- chronic or chronic administrations, considering the detrimental peripheral effect of the hormone, including feminization in males [28,32,33]. To evaluate the 12-week q.d. DHED eye drop treatments in this regard, serum E2 content together with the wet weights of the seminal vesicle (SV) and the anterior pituitary (AP) as typical estrogen-sensitive peripheral organs [48,49] were assessed (Table 1). Serum E2 quantitation was performed by utilizing our validated LC–MS/MS-based assay [56,57]. As seen in Table 1, blood E2 levels in the DHED-treated group were indistinguishable from those in the naïve and vehicle groups, respectively. Consistent with this outcome is the lack of wet weight increase in the SV and the AP, respectively, representing estrogen-sensitive peripheral organs. These findings show for the first time the inherent therapeutic safety of the DHED approach upon prolonged (12 weeks) daily treatments of the animals, as our earlier studies utilized only a 3-week q.d. treatment regimen [32,33].

### 3.2. Impact of Topical DHED Treatments on Visual Function Based on Targeted Proteomics in the Experimental Model of Glaucoma 

To delineate the neuroprotective effect of E2 delivered via its DHED prodrug to counteract OHT-induced vison decline in the selected animal model of glaucoma (Figure 2b,c) further, targeted retina proteomics of the DHED- versus vehicle-treated animals was also performed. In this experiment, we relied on our previously proposed panel of preclinical protein biomarkers of OHT-induced glaucomatous processes in the male BN rat retina [45]. The panel included apolipoprotein E (APOE), guanine nucleotide-binding protein G(o) subunit α (GNAO1), γ-synuclein (SNCG), cyclic nucleotide gated channel β-subunit (CNGB1), α-and β crystallins (CRYAA and CRYBB), Na+/K+-ATPase subunit α-1 (ATP1A1), hexokinase 2 (HK2), as well as adenosine triphosphate synthase F1 subunit β (ATP5F1B). The first three proteins were significantly upregulated by OHT, while the other six proteins (CNGB1, CRYAA, CRYBB, as well as ATP1A1, HK2, and ATP5F1B) were downregulated because of the elevated IOP [45].

**Figure 4:**
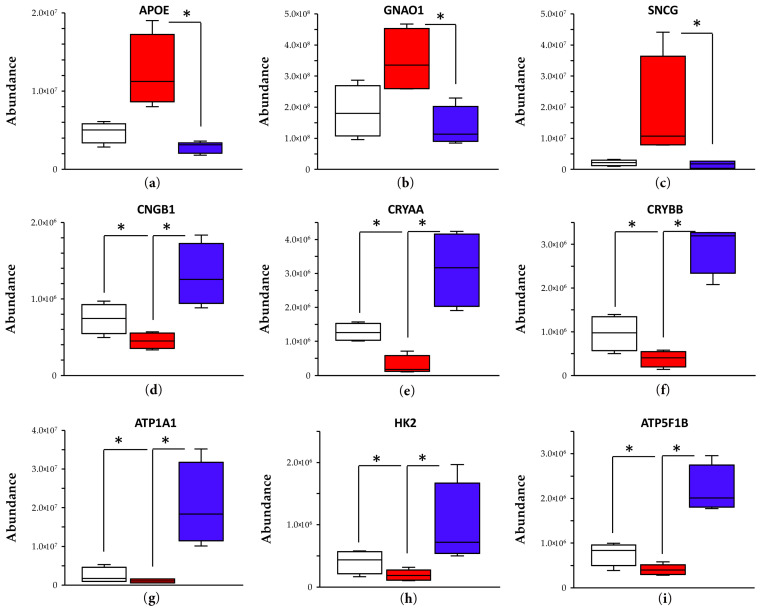
E2 delivered into the retina by topical DHED consistently counteracted the glaucomatous changes in the male BN rat retina based on LC–ESI-MS/MS-based quantitative survey by PRM and measuring on the expressions of selected proteins as surrogate endpoints: (**a**) cyclic nucleotide gated channel β-subunit (CNGB1); (**b**) α-crystalline A (CRYAA); (**c**) β-crystalline B (CRYBB); (**d**) apolipoprotein A (APOE); (**e**) guanine nucleotide-binding protein G(o) subunit α (GNAO1); (**f**) γ-synuclein (SNCG); (**g**) Na^+^/K^+^-ATPase subunit α-1 (ATP1A1); (**h**) hexokinase 2 (HK2); (**i**) adenosine triphosphate synthase F1 subunit β (ATP5F1B). Asterisks (*) denote statistically significant differences of OHT animals treated with DHED eye drops (blue boxes) from vehicle-treated OHT animals (red boxes) and, when applicable, normotensive naïve animals (open boxes) by one-way ANOVA followed by post hoc Tukey–Kramer tests (n = 4, *p* < 0.05).

The results of this targeted proteomics survey [33,45] are summarized in Figure 4. DHED-derived E2 consistently counteracted the glaucomatous dysregulations in the surveyed retina protein expressions (blue versus red boxes), and it completely “normalized” the OHT-upregulated proteins (APOE, GNAO1, and SNCG) to the levels seen in the age-matched normotensive, thus, healthy male rat retina (Figure 4, open boxes).

## 4. Discussion

Our goal in this study was to investigate whether retina-targeted delivery of E2 via its unique DHED bioprecursor prodrug [31,32,33] would halt OHT-induced retinal damage in a male rat model of glaucoma. We utilized a three-pronged phenotypic approach relying on behavioral assessments of vision, RGC, and axon survivals, as well as targeted retina proteomics to compare outcomes of the neuroprotective intervention under continuous OHT with those without the preventative treatment. 

It has been shown in various animal models of neurodegeneration impacting the central nervous system (CNS) that the main human estrogen, E2, acts as a broad-spectrum neuroprotectant [36,37,60]. The retina is part of the CNS [61]; therefore, it is not surprising that ocular and brain-related neurodegenerative processes share many pathogenic mechanisms [62]. In glaucoma, RGCs and their axons forming the optic nerve are gradually destroyed, leading to a reduction in visual field [11]. In animal models of the disease, E2 administration protected these vulnerable regions within the neural retina [28,63,64,65,66]. The hormone has also been proposed as an important contributor to the overall retina health [67,68]. Pharmacotherapeutic interventions relying on E2, however, are limited owing to side effects, including cardiovascular liability, initiation of certain cancers and feminization [28,35,36,46] that are associated with any currently known estrogen therapy. These caveats thus far have prevented the clinical realization of E2 as a neuroprotectant.

Previously, we have reported that our DHED prodrug’s distinguishing and unique feature is that it remains inert in the circulation and peripheral organs while rapidly metabolizing to E2 (Figure 1) at the site of action, in the present context in the retina, upon topical administration [31,32,33]. Accordingly, using the saline-induced OHT model of glaucoma [34] in male retired-breeder BN rats, animals were treated with DHED eye drops under continued, sustained OHT (Figure 2a) to survey the neuroprotective effect of DHED-derived E2 against OHT-triggered neurodegeneration. IOP elevation remained at approximately 45–50% of the baseline value during the 12-week observational period, as DHED did not have an IOP lowering effect. This is important, as it appears that IOP reduction is not sufficient to halt the disease progression as RGCs may continue to die even under controlled IOP [69,70,71]. Moreover, a significant portion of glaucoma patients are normotensive yet exhibit continued optic nerve damage with resultant loss of visual field [72,73]. Therefore, prevention of neuronal damage and loss in the glaucomatous retina is needed separately from the management of the IOP. Current therapies can only address IOP lowering while ocular neuroprotection remains an unmet medical need.

Indeed, despite sustained OHT (Figure 2a), a profound neuroprotection was observed in the DHED-treated group, based on the OMR-utilized behavioral assessments of vision (Figure 2b,c). In relation to the naïve, normotensive control, only around 10% loss was detected in VA in this group resulting in 50% higher acuity in relation to animals receiving the vehicle alone by the end of the 12-week observational period (Figure 2b). Alongside, approximately a 70% drop in CS at the given spatial frequency compared to the control was detected even after a 3-week sustained OHT without the targeted E2 treatment (vehicle alone), as shown in Figure 2c. In turn, animals receiving q.d. DHED eye drops exhibited a 2.5-fold higher CS than those in the vehicle-treated group. A similar observation was also found in our previous study using E2 eye drops [28]. While E2 itself was also devoid of an ocular antihypertensive effect, it provided a significant preservation of CS in the treated animals under sustained OHT conditions. 

Therefore, it is not surprising that the instillations of DHED prodrug eye drops produced comparable prevention of vison loss due to the DHED-to-E2 metabolism in the retina [31,32,33]. A profoundly different outcome was shown, however, in terms of peripheral exposure to the hormone upon using E2 eye drops. There was a significant increase in circulating E2 even after 3-week q.d. E2 eye drop treatments (414 ± 79 pg/mL [33] and 463 ± 29 pg/mL [28]). In turn, in the current study, blood E2 levels in the DHED group were indistinguishable from those of the control (naïve) and vehicle-treated groups, respectively, even after 12-week q.d. treatments (Table 1), implicating inherent therapeutic safety by our retina-targeted E2 delivery via the DHED prodrug. This is the first report to assess the peripheral impact of a 3-month daily topical DHED treatment in the animal model selected here. 

The measured visual functions in this group (Figure 2b,c) mirrored well the attenuation of OHT-induced apoptosis in the RGC layer. Elevated IOP has been shown to produce RGC atrophy with damage to axons occurring at the optic nerve head via a variety of mechanisms [11]. As such, in our model of glaucoma, in the vehicle-treated animals, indeed, there were around 60% fewer cells remaining than in the normotensive control by the end of the 12-week OHT exposure (Figure 3a). In contrast, in OHT rats receiving the neuroprotective treatment via the DHED-derived E2, there was a 2-fold higher number of RGCs present. Apparently, the neuroprotective intervention not only prevented significant RGC loss (Figure 3a), but the surviving RGCs also remained functional as reflected in the measured VA and CS in these animals (Figure 2b,c). These findings agree with our previous study showing that E2 eye drops significantly reduced OHT-induced apoptosis in the RGC layers [28]. Also consistent with RGC functional survival is the preservation of axons in the optic nerve cross section, as in the DHED group a 2-fold higher axon density was detected than that of the vehicle-treated group under continuous OHT (Figure 3b).

The above-described studies involving behavioral assessments of visual function (Figure 2b,c), as well as observation of RGCs survival (Figure 3a,b) under OHT with a focus on our neurotherapeutic intervention, were also complemented and corroborated by targeted retina proteomics. Our previously published proteomics studies in the selected animal model of glaucoma have uncovered OHT-associated dysregulations in the rat retina proteome [28,45]. By concentrating on proteins associated with neuroprotection, neurodegeneration, energy metabolism, and neuronal function using targeted proteomics, we selected a preclinical biomarker panel from proteins whose expressions were significantly affected by OHT compared to the normotensive control retinas (Figure 4 and ref. [45]). 

The panel included APOE, SNCG, and GNAO1, which were significantly upregulated by sustained IOP elevation (Figure 4). While APOE (Figure 4a) regulates cholesterol homeostasis, its overexpression under neuronal stress may cause axonal degradation by activating neurodegenerative phenotypes in astrocytes and microglia [74,75]. In fact, tissue-specific knockouts of APOE in retinal microglia alone have been shown to protect the RGCs against OHT-induced damage in another animal model of glaucoma [74]. Suppression of APOE also reduces glycoprotein galectin-3 (Lgals3), which has been shown to promote the survival of RGCs [74]. Lgals3 overexpression is also linked to elevated SNCG (Figure 4c) in the retina [75]. Therefore, SNCG-promoted expression of Lgals3 in optic nerve head astrocytes contributes to axonal loss. GNAO1, the α subunit of guanine nucleotide-binding proteins, plays a vital role in phototransduction [76]. Our finding (Figure 4b) that this protein was also upregulated by OHT [45] requires further exploration. However, as Figure 4a–c show, DHED-derived E2 not only counteracted the OHT-triggered upregulations of these proteins but also resulted in protein expression levels that were statistically not different from those of the normotensive, age-matched naïve control (open vs. blue boxes). 

The protein CNGB1 is predominantly expressed in the rod cyclic nucleotide-gated channel and plays a vital role in vision by means of membrane hyperpolarization [77]. In our experimental model of glaucoma, the abundance of this protein was significantly decreased without the neuroprotective intervention afforded by the DHED eye drops (Figure 4d). Crystallin proteins also showed significant downregulation by OHT (Figure 4e,f). While they are typically associated with the structure of the lens, crystallin proteins play signaling roles in retina health [78]. CRYAA is a heat shock protein that can promote resistance against the intrinsic apoptotic pathway by directly binding to the proapoptotic B-cell lymphoma-2 (BCL-2) family proteins Bim (BCL-2 protein 11) and Bax (BCL-2-associated X protein) [79]. These antiapoptotic effects align with a previous study that assessed the effects of E2 eye drops via TUNEL staining, which showed reduced double-stranded breaks in cells found in the RGC layer [28]. Similarly, CRYBB has been shown to promote resistance against retinal injury in both OHT and optic nerve crush models of the disease through various signaling pathways [80,81]. Intravitreal injection of CRYBB2 also protected the retinal nerve fiber thickness, RGC, and axon numbers despite OHT [80]. In our study, we found a significant upregulation of these neuroprotective proteins by DHED-derived E2 (Figure 4). In fact, upregulations of crystallins by administration of E2 eye drops have also been observed in the normotensive rat retina [58]. 

ATP1A1 is another neuroprotective protein that maintains neuronal membrane potential [82]. Under the effect of chronic OHT, ATP1A1 transcription and protein expression are reduced, thereby disrupting neuronal communication in the RGCs. Inhibitors of Na^+^/K^+^ transport turnover and, thus, the prevention of the protein’s removal provided neuroprotection against pressure-induced stress in vitro [82]. As both types of crystallins (CRYAA and CRYBB, Figure 4e,f) and ATP1A1 (Figure 4g) were significantly upregulated after topical application of DHED, these may have significantly contributed to the maintenance of retinal and optic nerve structure and function leading to the preservation of visual function even under OHT (Figure 2b,c and Figure 3a,b). Depending on the layer and cell type, increased glycolysis and oxidative phosphorylation (OXPHOS) favor cell survival by maintaining homeostasis and improving bioenergetics [83,84]. The RGCs are considered more glycolytic than the cells in the innermost retinal layers. HK2 expression was also reduced in OHT rats but was restored after topical application with DHED (Figure 4h). In the photoreceptors, HK2 is vital for regulating glucose metabolism, as photoreceptors lacking HK2 were more vulnerable to oxidative stress and nutrient deprivation resulting in a thinner outer nerve layer [85,86]. Furthermore, photoreceptors rely primarily on OXPHOS; therefore, an increased expression of the ATP synthase complex subunit ATP5F1B by E2-derived DHED (Figure 4i) suggests increased mitochondrial function [87]. Future studies could corroborate these findings by utilizing metabolic assays to assess oxygen consumption and glucose uptake. Altogether, our selected surrogate endpoints also indicated that topical DHED treatment provided retina-targeted remediation of the experimentally induced glaucoma in the hypertonic saline injection model using male BN retired-breeder rats.

## 5. Conclusions

Focusing on vision rescue as the goal of preclinical therapy development against glaucomatous neurodegeneration of the retina, we successfully demonstrated the potential value of the topically administered DHED, a prodrug of E2 that confined therapeutic effect to the site of action, based on OMR in a phenotypic model of glaucoma. Follow-up findings that assessed RGC and axon preservations were in complete agreement with the outcomes of the behavioral assessments of visual acuity and contrast sensitivity. In addition, the expression pattern of the chosen preclinical protein biomarkers associated with OHT-induced damage in the retina added valuable mechanistic insights into the evaluation of the phenotypic approach we employed.

## Figures and Tables

**Table 1:** The 12-week q.d. DHED eye drop treatments do not expose the periphery to the unwanted hormonal side effects of E2. Data are given as average ± standard deviation, n = 4 per group.

Group	E2 (pg/mL)	SV (mg)	AP (mg)
Naïve	2.3 ± 0.7	507.8 ± 50.1	5.2 ± 0.4
Vehicle	2.4 ± 0.6	501.0 ± 34.3	5.2 ± 0.6
DHED	2.2 ± 0.4	493.0 ± 15.8	5.2 ± 0.4

## Data Availability

Available upon request.

## Supplementary Materials

The following supporting information can be downloaded at: https://www.mdpi.com/article/10.3390/cells13131126/s1, Figure S1: Representative images of RGC labeling using RBPMS (upper panel, scale bar = 100 µm) as well as optic nerve axon labeling using PPD (lower panel, scale bar = 50 µm) from BN rats treated with either DHED or vehicle eye drops. Naïve (normotensive) rats served as control. Table S1: PRM setup for targeted proteomics to quantify fold changes of our panel of preclinical biomarkers (Figure 4) using the group comparison mode for native endogenous proteotypic peptides in Skyline.
