# Supplementary figures and images for: The Prodrug DHED Delivers 17β-Estradiol into the Retina for Protection of Retinal Ganglion Cells and Preservation of Visual Function in an Animal Model of Glaucoma
Source: Cells. 2024 Jun 29;13(13):1126. doi: 10.3390/cells13131126 (PMC11240555; doi:10.3390/cells13131126)

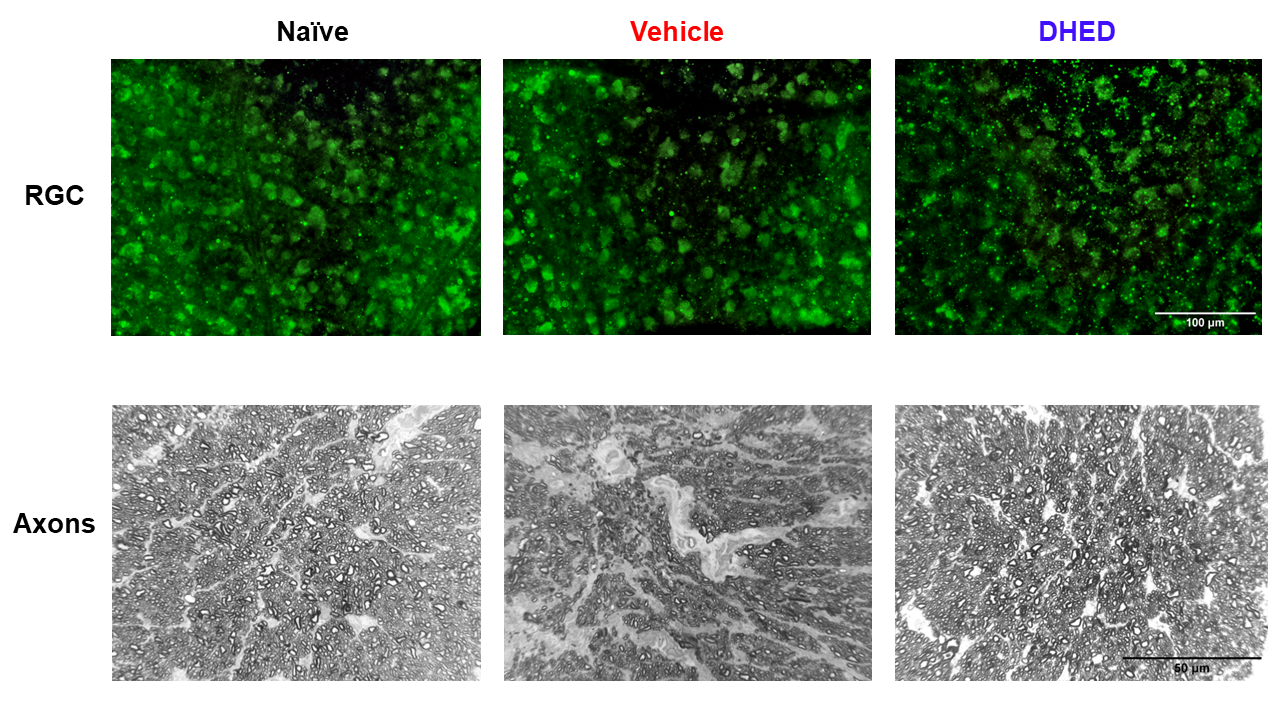

Supplement: Supplementary file 1 [file cells-13-01126-s001.zip › FigureS1_cells-3068067.tif]
